# Etiology and Outcome of Acute Liver Failure in Children—The Experience of a Single Tertiary Care Hospital from Romania

**DOI:** 10.3390/children7120282

**Published:** 2020-12-09

**Authors:** Alina Grama, Cornel Olimpiu Aldea, Lucia Burac, Dan Delean, Bogdan Bulata, Claudia Sirbe, Emanuela Duca, Dora Boghitoiu, Alexandra Coroleuca, Tudor Lucian Pop

**Affiliations:** 1 2nd Pediatric Clinic, University of Medicine and Pharmacy “Iuliu Hatieganu”, 400012 Cluj-Napoca, Romania; gramaalina29@gmail.com (A.G.); luciaburac@gmail.com (L.B.); claudia.sirbe@umfcluj.ro (C.S.); emanuelafloca@gmail.com (E.D.); 2 Center of Expertise in Pediatric Liver Rare Disorders, 2nd Pediatric Clinic, Emergency Clinic Hospital for Children, 400177 Cluj-Napoca, Romania; 3 University of Medicine and Pharmacy Carol Davila, 050474 Bucharest, Romania; dora.boghitoiu@umfcd.ro (D.B.); alexandracoroleuca10@gmail.com (A.C.); 4 Pediatric Nephrology, Dialysis and Toxicology Clinic, Emergency Clinic Hospital for Children, 400177 Cluj-Napoca, Romania; cornelaldea65@yahoo.com (C.O.A.); ddelean2003@yahoo.com (D.D.); iggbie@gmail.com (B.B.)

**Keywords:** acute liver failure, children, teenagers, etiology, outcome

## Abstract

Background: Acute liver failure (ALF) is a rare disease, associated with high mortality, despite optimal medical therapy without emergency liver transplantation. Knowing the possible cause of ALF plays a vital role in the management, as the child could benefit from effective specific therapies in emergencies. Methods: We have analyzed the etiology and outcome of ALF in children followed-up in a tertiary care hospital between January 2012–December 2018. The patients were grouped into different age categories: neonates (0–1 month), infants (1–12 months), children (1–14 years), and teenagers (14–18 years). Results: 97 children (46 males, 47.42%, the mean age of 7.66 ± 8.18 years) were admitted with ALF. The most important causes of ALF were in neonates and infants, infections (72.72%), and metabolic disorders (43.47%), in children and adolescents were the toxic causes (60% and 79.41%). The mortality rate was 31.95% (31 patients), mainly in ALF due to infections or metabolic disorders. Conclusions: In neonates and infants, the main causes of ALF were infections and metabolic diseases, while in older children and teenagers, were toxin-induced liver injuries. The mortality among neonates and infants was significantly higher than in other ages. Early recognition and immediate therapeutic intervention could improve the outcome of these patients.

## 1. Introduction

Acute liver failure (ALF) is a rare condition in pediatric pathology, with increased mortality (50%) despite optimal medical therapy in the absence of emergency liver transplantation [1,2,3]. ALF is a syndrome characterized by hepatic coagulopathy not-corrected by parenteral administration of vitamin K and the value of the INR (International Normalized Ratio) of 1.5–1.9 in the presence of hepatic encephalopathy or INR > 2.0 in the absence of hepatic encephalopathy in a child without the pre-existing liver disease [4,5,6]. The causes of ALF in children are different from those of the adult and vary according to the age of the child. This is due to age, limited defense resources, immaturity of organs or systems (in neonates and infants), or conditions specific to the period of childhood that can be extremely severe (metabolic disorders). If infectious causes and metabolic disorders are more common in neonates and infants, in older children and teenagers, besides viral etiologies, toxic causes, autoimmune disorders and Wilson’s disease (WD) are involved. In developed countries like the United States of America (USA), more than 45% of cases of ALF in children remain of unknown etiology [7,8,9]. Furthermore, ALF etiology in children varies according to geographical areas and socio-economic conditions. In developing countries, toxics are the first cause of ALF, while in countries with a low socio-economic level, infections, especially with hepatitis A (HAV) and B (HBV) virus, are the main causes [10,11]. Although ALF in children is a rare condition, it results in a high mortality rate in the absence of an emergency liver transplant.

Our study aimed to analyze the causes of ALF in children diagnosed and followed-up in a tertiary care hospital, to establish the particularities related to age and to compare our results with those from other regions of the World.

## 2. Materials and Methods

We analyzed the ALF patients followed-up in Gastroenterology, Toxicology, and Intensive Care Departments from the Emergency Clinical Hospital for Children, Cluj-Napoca, Romania, for 7 years (January 2012–December 2018). Our institution is the main hospital from the North-Western part of Romania accepting patients with severe liver disease and ALF, including toxic causes. We have included in the cohort 97 children (1–18 years) who met the Pediatric Acute Liver Failure (PALF) criteria for ALF, respectively patients without pre-existing liver disease who presented uncorrectable coagulopathy with INR > 1.5 in the presence of encephalopathy, or INR > 2 in the absence of hepatic encephalopathy [1,2,3]. The diagnosis and management of these cases were in accordance with international pediatric ALF guidelines, with the mention that Romania does not currently benefit from access to emergency liver transplant for neonates and infants.

We analyzed the demographic data, laboratory parameters (transaminases, bilirubin, gamma-glutamyl transferase, alkaline phosphatase, albumin, prothrombin time and INR, glycemia, complete blood count, and blood pH), and outcome of children with ALF (survival, liver transplantation, or death). The etiological diagnosis was based on clinical information, medical history, and laboratory parameters, including cultures. Viral etiology was confirmed with serological markers for HAV, HBV, hepatitis C virus (HCV), Cytomegalovirus (CMV), Epstein–Barr virus (EBV), Herpes Simplex virus (HSV), or echovirus. For immune disorders, we detected specific autoantibodies (anti-nuclear, anti-liver kidney microsome type 1, and anti-smooth muscle antibodies). Diagnosis of WD was based on the serum ceruloplasmin and 24 h urinary copper excretion levels. Inborn errors of metabolism (IEM) were detected through disease-specific metabolic panels or confirmed based on liver histopathology. Toxic etiology was established based on the positive history of mushroom or drug ingestion. Patients were divided into four groups based on age: neonate (0–1 month), infant (1–12 months), child (1–14 years), and adolescent (14–18 years). We excluded from the study oncology patients who developed toxic ALF after chemotherapy and those who developed ALF in the context of multiple organ failure. Children with HAV or hepatitis E virus (HEV) infection were not part of our study as they are hospitalized in the Infectious Diseases Hospital. We also excluded patients who did not fully meet the PALF criteria for ALF or had incomplete clinical or laboratory data.

The data obtained were included in a Microsoft Office Excel database and subsequently analyzed statistically, using descriptive statistics for variables with continuous distribution (means and standard deviations), t-Student test for statistical significance testing. The results were considered statistically significant at values of *p* < 0.05.

## 3. Results

Between 1 January 2012 and 31 December 2018, in the Emergency Clinic Hospital for Children Cluj-Napoca, Romania, 97 children (aged between 0 and 18 years, all from a Caucasian population) were followed for ALF secondary to ingestion of toxins (mushrooms or medicines), metabolic disorders, autoimmune diseases, or infections. 

The sex distribution of our cohort: 49 boys (50.51%) and 48 girls (49.48%). The mean age was 7.66 ± 8.18 years, higher in girls (8.03 ± 6.26 years) than in boys (6.10 ± 5.86 years, *p* = 0.049). The youngest was a new-born aged 3 days and the eldest a female teenager of 17 years and 11 months. We analyzed the etiology of ALF by four age categories: neonates (0–1 month), infants (1–12 months), children (1–14 years) and teenagers (14–18 years). Based on these, most cases of ALF were in children (36 cases, 37.11%), followed by infants (27 cases, 27.83%) then teenagers (25 cases, 25.77%) and neonates (9 cases, 9.27%) (Table 1).

Infections were the main cause of ALF in infants, while in children and teenagers the main causes were toxin-induced. In our cohort 11 patients had ALF of unknown etiology (11.34%) (Table 2).

In neonates, most patients developed ALF after infections: *Klebsiella pneumoniae*, *Pseudomonas aeruginosa*, CMV, or HSV type 1. Mortality in neonates was the highest in our cohort (due to infections, immune causes, and tyrosinemia). Only one neonate with ALF of unknown etiology received an emergency liver transplant in a pediatric liver transplant center, in Germany.

In infants, the main causes of ALF were infections and metabolic disorders. The viral infections were caused by CMV, EBV, HSV type 1, echovirus, or HBV. Among the bacterial agents involved we detected enterococcus and Gram-negative bacilli (*Klebsiella pneumoniae*, *Escherichia Coli*, or *Proteus*). Metabolic causes were represented by galactosemia, hereditary fructose intolerance, mitochondrial disorders, and tyrosinemia, the last two with fatal evolution in our study. The outcome of ALF in this age group was fatal in almost half of the cases. 

In children, toxic liver injury was the main cause of ALF, followed by infections, AIH and metabolic disorders. The etiology remained unknown in four cases. Toxic causes were drugs (albendazole, acetaminophen, isoniazid, valproic acid, or fluconazole) and wild mushrooms. There were two children with WD and one with a mitochondrial disorder. Further, there were six cases with ALF after infections (EBV, CMV, and Echovirus). Common infectious causes of ALF in children were EBV, CMV, and Echovirus. EBV has been involved in four cases of severe ALF: one with self-limiting evolution and three complicated with severe aplastic anemia, a combination that is very rare (0.03–0.2% of cases with aplastic anemia after acute hepatitis), severely affecting the disease course and management [12]. This specific complication significantly contributed to mortality in this age category (despite the immunosuppressive therapy), alongside metabolic disorders (WD and mitochondrial disorder) and ingestion of toxics (fluconazole and mushrooms).

Among teenagers, the causes of ALF were toxics, AIH (type 1 and type 2), WD, and infections. The toxicity was secondary to acetaminophen overdose (the minimum dose being 260 mg/kg, and the maximum 670 mg/kg), mushroom ingestion, albendazole, valproic acid, or colchicine use. In this age category, the disease course was fatal in three patients: one with mushroom intoxication, one case of suicidal colchicine ingestion and one with ALF of unknown etiology. Two cases of fulminant WD underwent an emergency liver transplant.

## 4. Discussion

ALF is associated with high mortality despite optimal medical therapy, especially if emergency liver transplantation is unavailable. The management of these cases requires a multidisciplinary team involving a pediatric hepatologist, critical care specialist, nephrologist, or liver transplant surgeon. 

Toxic liver injury is one of the most common causes of ALF in children (20–25% of cases) in Europe and the USA, secondary only to viral hepatitis. The most important toxics involved are mushrooms (Amanita Phalloides) and drugs (acetaminophen, valproic acid, or tuberculostatic therapy). Metabolic disorders and AIH are two other important causes of ALF in children, which can lead to an unfavorable outcome without prompt treatment. Despite the numerous investigations available, more than 45% of cases of ALF in children remain of unknown etiology [7,13,14,15,16,17].

Our study design is retrospective, analyzing ALF etiology in children admitted to the Emergency Clinic Hospital for Children Cluj-Napoca, the main Pediatric Centre in North-Western Romania. 

Overall, the toxic causes dominated the ALF etiology, followed by infections. Many toxic ALF cases in our study were due to wild mushroom consumption. Romania is well known for this foraging habit, but also for the loosely regulated access to “over the counter” drugs with hepatotoxic potential (acetaminophen and albendazole). 

We found an age-specific ALF etiology, with infections and metabolic causes predominating in neonates and infants, while toxics being the main etiology among older children. 

In neonates, the main etiologies were systemic infections with Gram-negative germs. These were mainly associated with intrapartum problems (difficult delivery/low Apgar score), low birth weight, prematurity, or invasive maneuvers (umbilical vein catheterization, invasive respiratory support). Further, congenital infection with CMV or HSV type 1 determined severe acute liver injury in neonates, which required aggressive treatment with ganciclovir or acyclovir. Mortality among these children was very high. We postulate low weight, prematurity, limited defense resources, and the severity of the conditions causing ALF in this age category (severe systemic infections or metabolic disorders) as risk factors for high mortality. Difficult access to emergency liver transplant at this age also plays a major role in our regional setting. 

Our results in this age category are similar to those in European medical literature. In countries such as Italy or the United Kingdom (UK) infections are the main cause of severe hepatic injury in neonates followed by metabolic disorders [4,18,19].

In infants (age 1–12 months), the main causes of severe liver injury were infections. The outcome was fatal in 22.23% of all infections despite therapeutic measures. In developing countries, there is a high prevalence of viral infections, in some regions these being the main cause of ALF in infants, with an important cause of morbidity and mortality [1,2,3,4,5]. Another important cause of severe liver injury in infants was represented by metabolic disorders. In our study, infants with hereditary fructose intolerance or galactosemia evolved favorably with a specific diet and supportive therapy. Early recognition of these disorders reduces the morbidity and mortality rates in affected infants [20]. Unfortunately, in our country, there is no extended screening for metabolic disorders, and, for this reason, the diagnosis of IEM is rarely established during the first month of life. This is one of the reasons for the diagnostic delay in disorders such as tyrosinemia or mitochondrial diseases. In our study frequently the diagnosis was established during the autopsy and no case benefited from liver transplantation. In almost a quarter of the infants from our cohort, the etiology of ALF remained unknown. 

IEM and infectious diseases represent the main causes of severe liver injury at this age in most of the developing countries [21]. The PALF study group found similar causes in a large study involving 127 children under 3 years of age with ALF. The main etiologies were metabolic disorders (galactosemia, tyrosinemia, mitochondrial disorders, respiratory chain defect, fatty acid oxidation defects, fructose intolerance, and urea cycle disorders), infections (HSV, adenovirus, CMV, EBV, and enterovirus). A smaller number of cases presented with ALF due to neonatal hemochromatosis, leukemia, and hemophagocytic syndrome [16]. 

The outcome was unfavorable in almost half of the infants included in our study. At this age group the liver transplantation is not possible to be performed in our country. In other studies, the mortality due to ALF in infants varies from 20% in the PALF study, to 34% in Portugal, and 42% in young infants (0–4 months) in the UK [15,16,22].

Additional measures to prevent infections, implementation of HSV screening for pregnant women, and extended neonatal screening tests for metabolic diseases could reduce the incidence and mortality due to ALF in neonates and infants.

In our cohort, most cases of ALF were in children (1–14 years), the leading cause being toxics. The most common toxics involved were mushrooms and drugs (acetaminophen and albendazole). Mushroom poisoning represents an important public health issue in our country because mushroom foraging is a popular activity in Romanian villages. Rainy periods in summer and autumn are associated with a spike in cases. In almost all cases who developed ALF after mushroom poisoning, the presenting symptoms (vomiting, diarrhea, and abdominal pain) were late-onset (more than 4 h after ingestion). These were followed by hepatic encephalopathy, jaundice, bleeding, or acute renal injury. Ingestion of wild mushrooms was the main cause of toxic hepatitis in the last 20 years in our hospital. In the last years, the number of children who developed ALF after mushroom poisoning decreased significantly, thus determining the reduction in the overall mortality [5]. 

Unfortunately, we have been seeing an increase in the number of children developing severe hepatotoxicity associated with the ingestion of drugs, albendazole being the most frequent. The use of this drug only in very well documented cases of parasitosis (hydatic cyst, aspergillosis, or severe parasite infections) could reduce the frequency of these ALF cases. 

In teenagers, the main cause of ALF was also the toxin-induced liver injury, especially drug-induced liver injury (DILI). Acetaminophen was often used voluntarily, with suicidal intent. One reason for this could be the unusual ease of access to this OTC drug in pharmacies. In the last years, the number of teenagers taking overdoses of drugs for suicide is increasing in our region. Like in our cohort, in the PALF study, the most common causes in children 3–18 years were acetaminophen overdose (21%) [16]. WD and AIH (type 1 and type 2) represent an important cause of ALF in teenagers. ALF caused by WD is a rare but extremely aggressive disorder, with a high risk of death in the absence of liver transplantation [1,4].

In a different study conducted in our clinic, we analyzed retrospectively all patients who developed ALF after mushrooms/drugs exposure during a period of eighteen years (January 2000–August 2018) in North-Western Romania. Of 123 cases with toxic ALF, 89 cases were secondary to mushrooms ingestion and only 34 cases were secondary to drugs. Before 2012, the leading cause of toxic ALF was mushroom poisoning, while since 2012 most cases were due to drug ingestion [5]. The mortality due to mushroom poisoning in our previous study was 51.68%, compared to only one case in the present study (16.67%), higher that in ALF due to drugs (6.89%). The prognostic factors for mortality in ALF due to mushroom poisoning were the presence of encephalopathy, acute kidney injury, and severity of liver injury at admission (high level of transaminases, bilirubin, and INR) [5,23].

The etiology of ALF in teenagers differs by country, according to geographical areas and socio-economic conditions. In developing countries from Asia, South America, and Africa, the main causes of ALF in children and teenagers are infections with HAV or HEV [24,25,26,27,28]. In developed countries, toxic-induced liver injury is the first cause of ALF in teenagers, as reported also by a study from King’s College Hospital, London [4,13,15]. Improvement in health education, more effective drug control policies and psychosocial prevention measures against teenage depression may be of benefit in decreasing toxic-induced ALF in children.

In our cohort, in 11.34% of cases, the etiology of ALF remained unknown. The number of ALF cases with an indeterminate cause in children varies in different studies: 31% of cases in King’s College Hospital study, 43% in a German study, while in the PALF group study the etiology remains unknown in 49% of the patients [13,15,29]. In Turkey, 18% of a cohort of 308 children with ALF were classified as cryptogenic hepatitis [30]. The factors that may influence the quick diagnosis of ALF etiology include prioritization of possible causes of ALF according to age, the availability of diagnostic methods or the possibility of performing emergency liver transplantation (as sometimes insufficient evaluation could be interrupted by death or liver transplant) [17]. In severe cases with a rapidly unfavorable course, liver transplantation should be a priority. Most likely, in countries with experienced liver transplantation centers where this procedure is readily available, it contributes to the higher incidence of unknown causes of ALF in their specific cohorts.

## 5. Conclusions

ALF is a rare condition incurring high mortality in the absence of prompt treatment. In some situations, the only therapeutic solution is emergency liver transplantation. The causes of ALF in children vary depending on age and are different from those of the adult. In our cohort, in neonates and infants, infectious and metabolic disorders were the most common causes of ALF. Patients in this age group had high mortality despite medical therapy, partly due to the inaccessibility to emergency liver transplantation at very young ages in our country. Toxic ALF and fulminant WD can have a severe disease course in older children and teenagers. Early recognition and immediate therapeutic intervention are important to improve outcomes in these patients.

## Figures and Tables

**Table 1:** Distribution of children hospitalized with acute liver failure (ALF) by age group and their outcome.

Age Group	Patients n (%)	Unfavorable Evolution	
		Deaths n (%)	Liver Transplant n (%)
Neonates (0–1 month)	9 (9.27%)	6 (66.67%)	1 (11.11%)
Infants (1–12 months)	27 (27.83%)	13 (48.14%)	0
Children (1–14 years)	36 (37.11%)	9 (25%)	0
Teenagers (14–18 years)	25 (25.77%)	3 (12%)	2 (8%)
Total	97 (100%)	31 (31.95%)	3 (3.09%)

**Table 2:** ALF etiology in children by age groups.

Etiology of ALF	Neonates	Infants	Children	Teenagers	Total
	(0–1 Month) n, % of Age Group	(1–12 Months) n, % of Age Group	(1–14 Years) n, % of Age Group	(14–18 Years) n, % of Age Group	(0–18 Years) n, %
**Toxin-induced**	**-**	**-**	**19 (52.78%)**	**16 (64%)**	**35 (36.08%)**
**DILI**	**-**	**-**	**14**	**15**	**29**
- acetaminophen	-	-	3	12	15
- albendazole	-	-	9	1	10
- isoniazid	-	-	1	-	1
- valproic acid	-	-	-	1	1
- fluconazole	-	-	1	-	1
- colchicine	-	-	**-**	1	1
**Mushrooms poisoning**	**-**	**-**	**5**	**1**	**6**
**Infectious diseases**	**5 (55.56%)**	**15 (55.56%)**	**6 (16.67%)**	**1 (4%)**	**27 (27.83%)**
**Viral infection**	**3**	**9**	**6**	**1**	**19**
- Cytomegalovirus	1	3	1	-	5
- Herpes simplex virus type 1	2	2	-	-	4
- Epstein–Barr virus	**-**	2	4	1	7
- Hepatitis B virus	**-**	1	-	-	1
- Enterovirus	**-**	1	1	-	2
**Bacterial infection**	**2**	**6**	**-**	**-**	**8**
- *Klebsiella pneumoniae*	1	1	-	-	2
- *Pseudomonas aeruginosa*	1	1	-	-	2
- *Escherichia coli*	-	2	-	-	2
- *Enterococcus*	-	2	-	-	2
**Metabolic diseases**	**1 (11.11%)**	**7 (25.92%)**	**3 (8.33%)**	**3 (12%)**	**14 (14.43%)**
**IEM**	**1**	**7**	**1**	**-**	**8**
- galactosemia	-	2	-	-	2
- hereditary fructose intolerance	-	2	-	-	2
- mitochondrial disorders	-	2	1	-	3
- tyrosinemia	1	1	-	-	2
**Wilson Disease**	**-**	**-**	**2**	**4**	**6**
**Immune diseases**	**2 (22.22%)**	**-**	**4 (11.11%)**	**4 (16%)**	**10 (10.33%)**
AIH	-	-	4	4	8
- type 1 AIH	-	-	3	3	6
- type 2 AIH	-	-	1	1	2
Neonatal lupus	1	-	-	-	-
Gestational alloimmune disease	1	-	-	-	-
**Unknown**	**1 (11.11%)**	**5 (18.52%)**	**4 (11.11%)**	**1 (4%)**	**11 (11.34%)**
Total	9	27	36	25	97

ALF—Acute Liver Failure; AIH—Autoimmune hepatitis, IEM—Inborn errors of metabolism; DILI—Drug-induced Liver Injury.
